# Gestational exposure to organochlorine compounds and metals and infant birth weight: effect modification by maternal hardships

**DOI:** 10.1186/s12940-024-01095-x

**Published:** 2024-07-01

**Authors:** Janice M. Y. Hu, Tye E. Arbuckle, Patricia A. Janssen, Bruce P. Lanphear, Joshua D. Alampi, Joseph M. Braun, Amanda J. MacFarlane, Aimin Chen, Lawrence C. McCandless

**Affiliations:** 1 Environmental Health Science and Research Bureau, Healthy Environments and Consumer Safety Branch, Health Canada, 101 Tunney’s Pasture Driveway, Ottawa, ON K1A 0K9 Canada; 2 Faculty of Health Sciences, Simon Fraser University, Burnaby, BC V5A 1S6 Canada; 3 School of Population and Public Health, University of British Columbia, Vancouver, BC V6T 1Z4 Canada; 4 Department of Epidemiology, Brown University, Providence, RI USA; 5 Texas A&M Agriculture, Food and Nutrition Evidence Center, Fort Worth, TX USA; 6 Department of Biostatistics, Epidemiology and Informatics, University of Pennsylvania Perelman School of Medicine, Philadelphia, PA USA

**Keywords:** Fetal growth, Birth weight, Maternal hardships, Metals, Organochlorine compounds, Two-hits, Chemical mixtures, Elastic net, Effect modifications, Interactions

## Abstract

**Background:**

Gestational exposure to toxic environmental chemicals and maternal social hardships are individually associated with impaired fetal growth, but it is unclear whether the effects of environmental chemical exposure on infant birth weight are modified by maternal hardships.

**Methods:**

We used data from the Maternal-Infant Research on Environmental Chemicals (MIREC) Study, a pan-Canadian cohort of 1982 pregnant females enrolled between 2008 and 2011. We quantified eleven environmental chemical concentrations from two chemical classes – six organochlorine compounds (OCs) and five metals – that were detected in ≥ 70% of blood samples collected during the first trimester. We examined fetal growth using birth weight adjusted for gestational age and assessed nine maternal hardships by questionnaire. Each maternal hardship variable was dichotomized to indicate whether the females experienced the hardship. In our analysis, we used elastic net to select the environmental chemicals, maternal hardships, and 2-way interactions between maternal hardships and environmental chemicals that were most predictive of birth weight. Next, we obtained effect estimates using multiple linear regression, and plotted the relationships by hardship status for visual interpretation.

**Results:**

Elastic net selected *trans*-nonachlor, lead, low educational status, racially minoritized background, and low supplemental folic acid intake. All were inversely associated with birth weight. Elastic net also selected interaction terms. Among those with increasing environmental chemical exposures and reported hardships, we observed stronger negative associations and a few positive associations. For example, every two-fold increase in lead concentrations was more strongly associated with reduced infant birth weight among participants with low educational status (*β* = -100 g (g); 95% confidence interval (CI): -215, 16), than those with higher educational status (*β* = -34 g; 95% CI: -63, -3). In contrast, every two-fold increase in mercury concentrations was associated with slightly higher birth weight among participants with low educational status (*β* = 23 g; 95% CI: -25, 71) compared to those with higher educational status (*β* = -9 g; 95% CI: -24, 6).

**Conclusions:**

Our findings suggest that maternal hardships can modify the associations of gestational exposure to some OCs and metals with infant birth weight.

**Supplementary Information:**

The online version contains supplementary material available at 10.1186/s12940-024-01095-x.

## Background

Fetal growth is a complex process impacted by various factors including psychosocial stressors, toxic chemicals, nutrition, placental function, and the actions of many intrauterine hormones and growth factors [1]. Fetal growth restriction, commonly assessed by infant birth weight for a given gestational age [2], occurs when the fetus does not reach its intrauterine potential for growth and development [3]. It is an important determinant for the risk of infant mortality and the development of chronic diseases [2].

Recent studies have found that impaired fetal growth is associated with gestational exposure to toxic environmental chemicals, which are ubiquitous in our environment, food sources and personal care products [1, 4, 5]. However, few have reported contradictory results [1, 6]. In our previous work, we reported associations of two classes of environmental chemicals – metals and organochlorine compounds (OCs) – with reduced infant birth weight [7]. We found that first trimester blood concentrations of *trans*-nonachlor, an organochlorine compound (OC), and lead (Pb), a toxic metal, were inversely associated with birth weight. Every twofold increase in *trans*-nonachlor and Pb concentrations was associated with reduced birth weight by an average of 38 g (g) (95% confidence interval (CI): -67, -10) and 39 g (95% CI: -69, -9), respectively. Our study, however, did not consider the effects of non-chemical stressors such as maternal hardships [8–10].

Recent research by Goin et al. (2021) reported that maternal hardships such as stressful life events, food insecurity, living alone and facing unplanned pregnancy can interact in pairwise fashion to adversely affect fetal growth [11], potentially via behavioral, physiological or immunological mechanisms [11–14]. These 2-way interactions observed are supported by Knudson’s “two-hit” hypothesis, where it was found that two gene mutations are needed for retinoblastoma, a childhood cancer of the retina, to develop [13]. The major implication of this “two-hit” hypothesis is that two distinct gestational stressors are needed to affect an outcome. Thus, gestational exposure to environmental chemicals and maternal hardships may interact and jointly influence fetal growth.

While there is robust evidence of an association between either chemical exposures or maternal hardships and fetal growth, there is a paucity of research on the joint associations of chemical exposures and maternal hardship on fetal growth. Aker et al. (2020) found evidence, among the 752 Puerto Rican females from the Puerto Rico Testsite for Exploring Contamination Threats (PROTECT) cohort, that life event score or stress interacted with bisphenol-S and triclocarban exposures to modify their associations with gestational length [15]. Although Aker et al. (2020) did not examine metals and OCs specifically, their observation supports further exploration of maternal hardships as an effect modifier in the associations between environmental chemicals and fetal growth.

We assessed the independent associations of nine maternal hardships including self-reported belonging to a racially minoritized group, immigrant status, financial strain, low supplemental folic acid intake, low educational status, living status, lone parenthood, experiencing chronic diseases, and being a student, on birth weight and the potential modifying effects of these maternal hardships on the associations between OCs and metals and birth weight among a cohort of 1982 Canadian females and their infants who participated in the Maternal-Infant Research on Environmental Chemicals (MIREC) Study.

## Methods

### Study participants

We analyzed data from the MIREC Study, a cohort study of 1982 pregnant females recruited between 2008 and 2011 from ten Canadian cities. The goal of the MIREC Study was to obtain Canadian biomonitoring data on pregnant females and examine associations between prenatal exposure to environmental chemicals with pregnancy and child health outcomes [16]. Detailed information on demographic and lifestyle factors were collected from questionnaires administered at recruitment in the first trimester. Eligibility criteria and exclusions are described in Arbuckle et al. (2013) [16]. For the present study, we included females who had complete socio-demographic information, provided biological samples during the first trimester of the pregnancy, and delivered singleton live births. Participants with missing information were excluded from the analysis (Supplemental Figure S1).

### Fetal growth measurement

To examine fetal growth, we considered infant birth weight adjusted for gestational age (GA) [17, 18]. We used cubic splines to allow infant birth weight curves to vary across gestation in a smooth manner [18]. Both infant birth weight, measured in grams (g), and GA, measured in weeks, were abstracted from the medical records and examined as continuous variables.

### Biomarkers of prenatal environmental chemical exposure

We measured environmental chemical exposures using biomarkers in blood samples collected from MIREC participants during the first trimester of pregnancy. Biomarker analysis was conducted at the Toxicology Laboratory of the Institut national de santé publique du Québec (INSPQ) using previously described methodology [19–21]. A brief description of the laboratory analysis method can be found in Supplemental Appendix A.

In our previous analysis, we reported associations between two classes of environmental chemicals – metals and OCs – and reduced infant birth weight [7]. Hence, in this study, we limited our analysis to OCs and metals only. We retained biomarkers that were detected in at least 70% of the samples [22]. These included six plasma OCs (polychlorinated biphenyl (PCB) 118, PCB 180, Aroclor 1260 (consists of PCB 138 and 153), dichlorodiphenyldichloroethylene (DDE), oxychlordane, and *trans*-nonachlor) and five whole blood metals or metalloids (arsenic (As), cadmium (Cd), mercury (Hg), manganese (Mn), and lead (Pb)).

We imputed measurements below the limit of detection (LOD) using the single imputation “fill-in” approach where the log_2_ chemical concentrations lower than the LOD were randomly sampled from a truncated lognormal distribution with mean and standard deviation (SD) estimated from the observed data [22]. To account for individual-level variability in lipid levels, we standardized OC concentrations by total plasma lipid concentrations and expressed them in units of ng/g lipids [23]. Standardization was only applied when presenting the descriptive statistics on the environmental chemical concentrations. Otherwise, total lipids was included as a covariate in regression models [23, 24]. Due to the highly correlated nature of PCB 153 and PCB 138, INSPQ summed and multiplied them by a factor of 5.2 to create Aroclor 1260 [25]. To reduce the potential influence of outliers due to the right skewed distributions of chemical concentrations, the concentrations were log_2_-transformed before inclusion in the models. The transformed unit indicates a two-fold increase in concentrations. Furthermore, before creating the interaction terms with maternal hardships to examine their joint associations, the log_2_-transformed chemical concentrations were median-centered to improve interpretation and decrease the multicollinearity effects, which would affect model convergence and inflate the standard errors [26].

### Maternal social hardships

We selected maternal social hardship variables based on factors related to social determinants of health [27]. We used a questionnaire administered at baseline [16] to capture responses that signal indicators of hardships directly affecting the study participants. The eight maternal hardships included self-reported belonging to a racial and ethnic minoritized group [8, 28], immigrant status [28, 29], financial strain [9, 28], low supplemental folic acid intake [30, 31], low educational status [8, 9], living status [32, 33], lone parenthood [34, 35], and experiencing chronic diseases [36, 37]. Furthermore, we also examined whether being a student is a potential maternal hardship. Each hardship variable was dichotomized to indicate whether a study participant experienced the hardship or not.

Race has definitions ranging from biological to social and its operationalization as a variable in research has been debated [38]. Race or having a racially minoritized background, as reported here, is considered a social construct that reflects lived experiences of systematic discrimination [39]. We classified participants who reported their racial or ethnicity status as non-white as belonging in a racially minoritized group [40].

Participants who reported their birth country as countries other than Canada were considered immigrants and faced hardship associated with being immigrants. Participants were classified as having financial strain if their reported annual household income was below $20,000, which is approximately the 2008 Canada poverty line for a two-person household [41]. Folate is a key pregnancy nutrient and pregnant individuals with hardship of food insecurity are more likely to have inadequate folic acid supplementation [30, 31]. We classified participants with daily folic acid supplementation levels below 400 µg per day as low supplemental folic acid intake given that they did not meet the recommended intakes for females at low-risk for a neural tube defect-affected pregnancy [42]. Supplemental folic acid intake was assessed via a structured survey conducted at 16-weeks’ gestation where participants were asked to list all the supplements they took in the past 30 days. The product information and intake frequency were used to calculate daily total folic acid consumption from supplements [43]. However, when supplement information was missing from this 30-day recall form, we then estimated the supplement information using the 24-h recall form (also completed at 16-weeks’ gestation) (*n* = 41; 2%) or if necessary, the baseline questionnaire, completed between 6- and 13-weeks’ gestation, where participants were asked to list all the supplements they had taken within the past 3 months (*n* = 462; 23%).

Participants who reported their highest educational level achieved as high school diploma or less were classified as having low educational status. For living status, participants who reported not living with a roommate, spouse, partner, or parents were classified as living alone. Participants with marital status as single (i.e., not married or not with the same partner for 1 year or more) were classified as having a hardship of lone parenthood. Participants who responded affirmatively to “Do you have any chronic medical condition(s)?” were classified as having chronic disease(s). Here, chronic medical condition was defined as any condition that treatment can manage but not cure. Examples of such conditions include high blood pressure, diabetes, and other pre-existing conditions prior to pregnancy such as asthma, depression, arthritis, heart conditions, and psoriasis. Lastly, we also examined whether being a student is a potential maternal hardship. Participants who answered yes to the question “are you currently attending school?” were classified as being current students.

### Covariates

We created a directed acyclic graph (Figure S2) to identify predictors of infant birth weight and factors associated with both environmental chemical exposure and infant birth weight. Additionally, we identified predictors of maternal hardships and infant birth weight. The following, derived from the baseline questionnaire, were examined: maternal age (under 25, ≥ 25 to < 30, ≥ 30 to < 35, ≥ 35 to < 40, and ≥ 40), race and ethnicity (white, non-white), education (high school diploma or less, college or trade school diploma, undergraduate university degree, and graduate university degree), cigarette smoking status (never, current, former, and quit during pregnancy), parity (0, 1, 2, ≥ 3), infant sex (male and female) and pre-pregnancy body mass index (BMI) (underweight, normal, overweight, and obese). We adjusted for the non-linear effect of gestational age (GA) as a covariate on birth weight using the cubic spline approach [18]. For OC models, we additionally adjusted for plasma lipid concentrations. For models assessing maternal hardship belonging to a racially minoritized group, we did not adjust for maternal race and ethnicity. For models assessing maternal hardship low educational status or low income, we did not adjust for maternal education.

### Analytical approach

We first tabulated participant characteristics, examined the relations between maternal hardship variables using Cramer’s V correlation coefficients, and calculated geometric means and percentiles of the environmental chemicals. We then proceeded to examine the moderating influence of maternal hardships on the relationship between OC and metal exposures and birth weight by using elastic net regression to choose which variables to include in our models.

Elastic net is a machine learning method that is built on conventional regression with an added penalty term [44, 45]. The penalty term biased the estimates to reduce overfitting and improve prediction on data not used in the model fitting procedure. There are two penalty parameters (i.e., ɑ and λ) that can be tuned to produce the best performing model. The quantity ɑ adjusts the balance between lasso and ridge penalty and has a range of 0 to 1. When ɑ is 1, the model is a lasso model and when ɑ is 0, the model is a ridge regression model. Furthermore, when ɑ is closer to 1, the elastic net model will focus more on selection and when the ɑ is closer to 0, the model can better handle multicollinearity. The second tuning parameter λ controls the magnitude of the penalty where smaller λ leads to estimates that are closer to those of conventional regression method. To determine the optimal degrees of penalization, we used cross-validation and tested the models over a grid of ɑ and λ sequences.

We used elastic net for variable selection purposes in order to reduce multicollinearity and to ensure that only important variables and interaction terms are included in the model, thus potentially reducing the probability of type I errors. Eventhough elastic net can account for multicollinearity, we created separate models for the OC compounds and the metals to avoid the curse of dimensionality, which will result in decreased statistical power to detect a true effect [44]. In each model, the dependent variable was birth weight adjusted for gestational age, and the predictor variables included all environmental chemicals in the same chemical class, all the maternal hardships, and the interaction terms between the environmental chemicals and the hardships (e.g., Pb x low education). The covariates and the cubic spline gestational age were adjusted in the elastic net regression model as unpenalized variables.

For parameter estimation and calculating 95% CIs, it has been recommended that penalized regression, such as elastic net regression, be used to select the model first and then the selected model be used in an unpenalized regression (i.e., ordinary least squares (OLS) regression models) to quantify the associations of gestational exposures to individual environmental chemicals with infant birth weight, while adjusting for covariates [45, 46]. This utilizes the predictive power of the overall modeling procedure while maintaining the interpretability of the individual estimates. We therefore fitted the subset of variables and the interaction terms selected and inferred to be important by elastic net in OLS regression models. The selected environmental chemicals and maternal hardships were entered into the OLS regression models individually and adjusted for covariates. The selected interaction terms were also entered into OLS regression models that included both the main effects and the interaction terms and adjusted for covariates. We examined the associations based on hardship levels, screened for suggestive associations with *p*-values of 0.1 and 0.05, and visualized the relationships graphically using interaction plots with 95% CI bands.

Furthermore, using Cook’s distance [47], we identified and removed one influential outlier that negatively affected the models. All analyses were conducted using Microsoft R Open version 3.5.1 and elastic net models were fitted using the *glmnet* package [48].

#### Supplemental analyses

We conducted additional analyses using first trimester plasma total folate concentrations instead of supplemental folic acid intake. Plasma total folate, a biomarker of folate status, specifically of recent folate intake, was measured by LC–MS/MS, as previously described [43]. Currently, no cutoff values for either low or high concentrations or a reference range for plasma total folate have been identified for fetal growth [49] but 25.5 nmol/L was estimated as a potential cut-off for neural tube defects (NTD) risk reduction [50]. Among the MIREC participants, less than 0.5% (*n* = 8) had a plasma total folate concentration < 25.5 nmol/L, while approximately 5% (*n* = 104) reported a low total daily supplemental folic acid intake of < 400 μg/day. Therefore, to allow for easier comparison of the low plasma total folate and the low supplemental folic acid intake effects and to mitigate issues with low sample size, we selected the 5th percentile of plasma total folate (51.5 nmol/L) as the cutoff value to indicate low folate status.

GA is a potential mediator in the relationship between environmental chemical exposures and birth weight [51]. To rule out any bias or over-adjustment in our models, we conducted additional analyses that excluded GA as a covariate in the models. Lastly, we summed the number of hardships to examine the association between cumulative hardship and birth weight.

## Results

### Descriptive statistics

We included a total of 1982 MIREC participants in the analysis (Figure S1). Most of the MIREC participants were white (82%), over 30 years of age (70%) with at least an undergraduate degree or higher (63%), never smoked (61%) and had normal BMI (61%). Forty-four percent were nulliparous and 53% had male infants. Maternal characteristics associated with lower birth weight included being non-white, older age, lower educational status, smoking, underweight BMI, and nulliparity. Female infants weighed on average 100 g lower at birth compared to males (Table 1).

**Table 1:** Participant sociodemographic characteristics and mean birth weight (grams) among MIREC study participants in Canada, 2008–2011

	**n (%)**^**a**^	**Birth weight (g)** **Mean (SD)**
Total	1982 (100)	3452 (532)
Race and ethnicity		
White	1517 (82)	3479 (529)
Others	339 (18)	3334 (529)
Age		
≤ 24	117 (6)	3404 (617)
25–29	443 (24)	3483 (505)
30–34	655 (36)	3452 (495)
35–39	502 (27)	3444 (570)
40 +	127 (7)	3428 (561)
Education levels		
High school diploma or less	160 (9)	3381 (571)
Some college, trade school, or college diploma	538 (29)	3444 (546)
Undergraduate degree	681 (37)	3486 (512)
Graduate degree	475 (26)	3435 (528)
Household income		
≤ $20,000	72 (4)	3348 (515)
$20,001—$40,000	152 (9)	3445 (572)
$40,001—$60,000	187 (11)	3458 (543)
$60,001—$80,000	286 (16)	3457 (530)
$80,001—$100,000	358 (20)	3440 (531)
> $100,000	715 (40)	3475 (516)
Smoking status		
Never	1137 (61)	3442 (520)
Current	105 (6)	3366 (657)
Former	498 (27)	3496 (517)
Quit during Pregnancy	115 (6)	3432 (569)
Parity		
0	812 (44)	3407 (546)
1	751 (40)	3496 (508)
2	221 (12)	3469 (559)
3 +	72 (4)	3454 (502)
Pre-pregnancy BMI		
Underweight	49 (3)	3302 (503)
Normal	1040 (61)	3429 (496)
Overweight	371 (22)	3515 (521)
Obese	258 (15)	3466 (647)
Infant Sex		
Male	974 (53)	3500 (534)
Female	877 (47)	3399 (524)

^a^numbers may not sum up to total due to missing data

From the Cramer’s V correlation coefficient plot (Figure S3), we observed low to moderate correlations (0 to 0.5) between the maternal hardship variables. The most commonly experienced maternal hardships were having a chronic disease (25%), being an immigrant (19%) and belonging to a racially minoritized group (18%). The least common maternal hardship was living alone (2%). All hardships, except for being a student, were associated with lower birth weight (Table 2).

**Table 2:** Participant hardship characteristics and mean birth weight (grams) among MIREC study participants in Canada, 2008–2011

	**n (%)**^**a**^	**Birth weight (g)** **Mean (SD)**
Total	1982 (100)	3452 (532)
Racially minoritized background		
Yes	339 (18)	3333 (529)
No	1517 (82)	3479 (529)
Low income		
Yes	72 (4)	3348 (515)
No	1698 (96)	3460 (529)
Low education		
Yes	160 (9)	3380 (571)
No	1649 (91)	3458 (528)
Lone parenthood		
Yes	87 (5)	3398 (626)
No	1769 (95)	3455 (527)
Living alone		
Yes	36 (2)	3384 (645)
No	1820 (98)	3454 (529)
Current student		
Yes	201 (10)	3461 (484)
No	1763 (90)	3451 (537)
Immigrant		
Yes	370 (19)	3379 (549)
No	1612 (81)	3469 (526)
Low supplemental folic acid intake (< 400$u$g/day)		
Yes	104 (5)	3361 (523)
No	1878 (95)	3457 (532)
Having chronic illnesses		
Yes	491 (25)	3404 (579)
No	1476 (75)	3467 (514)

^a^numbers may not sum up to total due to missing data

More than half of the participants (57%) reported having at least one maternal hardship(s) (Table S1). The most common hardships occurring together were immigrants with racially minoritized background at 10%, followed by low education with racially minoritized background, and immigrants who were students, both at 3% (Table S2). Furthermore, we detected most chemicals in over 90% of study participants, with the exception of PCB 118 and *trans*-nonachlor with 74% and 84% detection rates, respectively (Table S3).

### Variables and interaction terms that were selected in the elastic net models of infant birth weight

**Fig. 1:**
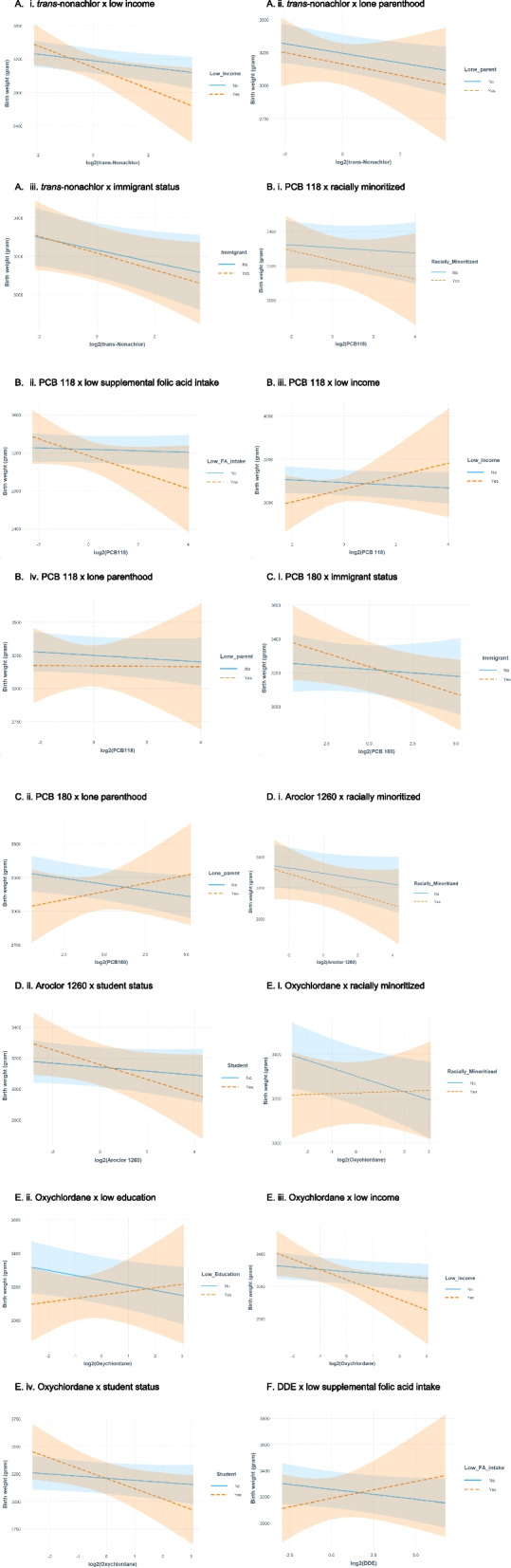
Interaction plots as selected by elastic net showing the differences in mean birth weight (grams) and 95% confidence intervals (shaded bands) associated with exposures to organochlorine compounds during the first trimester across different levels of maternal hardships. **A** Interaction plots for *trans*-nonachlor. **B** Interaction plots for PCB 118. **C** Interaction plots for PCB 180. **D** Interaction plots for Aroclor 1260. **E** Interaction plots for Oxychlordane. **F** Interaction plot for DDE

In the OC model, *trans*-nonachlor and three maternal hardship variables (i.e., low educational status, racially minoritized background, and low supplemental folic acid intake) were selected and inferred as important contributors in the elastic net regression of infant birth weight model. Elastic net identified sixteen interaction terms as important (Fig. 1).

**Fig. 2:**
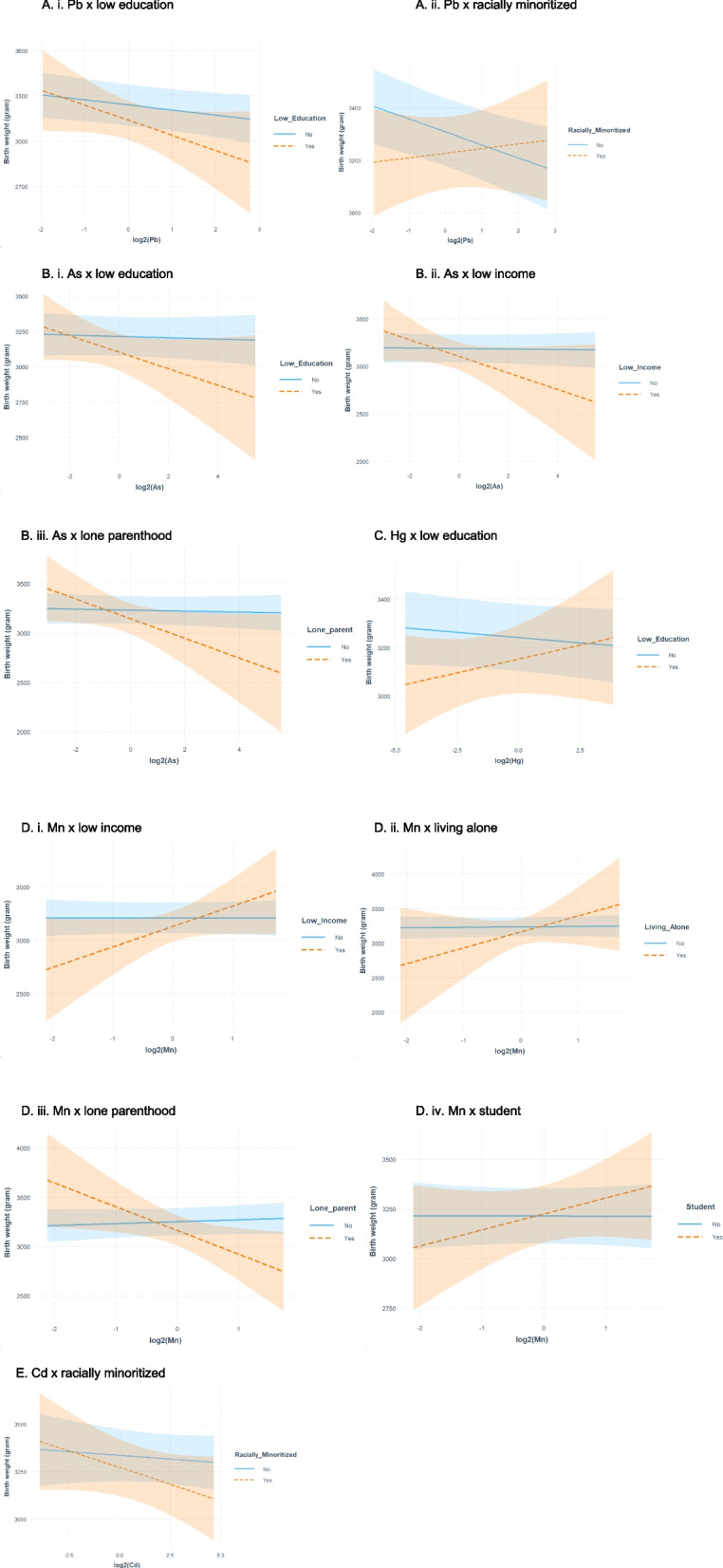
Interaction plots as selected by elastic net showing the differences in mean birth weight (grams) and 95% confidence intervals (shaded bands) associated with exposures to metals during the first trimester across different levels of maternal hardships. **A** Interaction plots for Pb. **B** Interaction plots for As. **C** Interaction plot for Hg. **D** Interaction plots for Mn. **E** Interaction plot for Cd

In the metal model, Pb and the same three maternal hardship variables (i.e., low educational status, racially minoritized background, and low supplemental folic acid intake) were selected. Elastic net identified eleven interaction terms as important (Fig. 2).

### Independent associations between environmental chemical exposure and infant birth weight

Our elastic net variable selection results agreed with those of Bayesian Kernel Machine Regression method from our previous study [7]. Both mixture methods indicated the importance of *trans*-nonachlor and Pb exposures, which were negatively associated with infant birth weight (Table 3). Every doubling of *trans*-nonachlor and Pb concentrations corresponded to a 37 g (95% CI: -65, -8) and a 38 g (95% CI: -68, -8) reduction in birth weight, respectively.

**Table 3:** Differences in mean birth weight (grams) and 95% CI associated with exposure to log_2_-chemicals and maternal hardships, as selected by elastic net, among the MIREC study participants in Canada, 2008–2011

	Unadjusted	Adjusted for covariates^a^
**Organochlorine Compounds (OCs)**		
* trans*-Nonachlor	-66 (-96, -35)	-37 (-65, -8)
**Metals**		
Lead	-56 (-92, -21)	-38 (-68, -8)
**Maternal Hardships**		
Racially minoritized background	-145 (-208, -83)	-78 (-130, -26)
Low education	-78 (-164, 9)	-103 (-185, -21)
Low supplemental folic acid intake (< 400$u$g/day)	-97 (-205, 12)	-61 (-152, 31)

^a^All models were adjusted for gestational age, race and ethnicity, education, age, BMI, smoking, parity, and baby sex. The OC models also adjusted for total lipids. Models assessing hardship of low education and racially minoritized background did not adjust for education and race and ethnicity, respectively, as a covariate

### Independent associations between maternal hardships and infant birth weight

 We found that all three hardships selected by elastic net (i.e., low educational status, racially minoritized background and low supplemental folic acid intake) had negative associations with birth weight (Table 3). Infants born to females with a high school diploma or less had lower birth weight ($\beta$ = -103 g; 95% CI: -185, -21) compared to those born to more educated females. Infants born to females with racially minoritized background had lower birth weight ($\beta$ = -78 g; 95% CI: -130, -26) compared to those born to white females. Lastly, infants born to females with low supplemental folic acid intake had lower birth weight ($\beta$ = -61 g; 95% CI: -152, 31) compared to those born to females who met or exceeded recommended folic acid intakes. 

### Regression analysis of the modifying effect of maternal social hardships on the relationship between environmental chemical exposure and infant birth weight

We found that maternal hardships can modify the strength of the relationships (i.e., steeper negative slope and lower birth weight) or the direction of the relationships (i.e., positive slope and higher birth weight) between biomarker of exposure to OCs and metals and birth weight. Although the 95% CIs were imprecise, some associations showed statistical significance at the 0.05 level (Tables 4 and 5).

**Table 4:** The associations between exposure to organochlorines (2-fold increase) and birth weight (grams) by maternal hardships among the MIREC study participants in Canada, 2008–2011

	**Low education**		**Low income**		**Immigrant**		**Low supplemental folic acid intake (< 400** $\mu$**g/day)**		**Lone parenthood**		**Racially minoritized background**		**Student**	
	**Yes** ***n* = 160** **(9%)**	**No** ***n* = 1649** **(91%)**	**Yes** ***n* = 72** **(4%)**	**No** ***n* = 1698** **(96%)**	**Yes** ***n* = 370** **(19%)**	**No** ***n* = 1612** **(81%)**	**Yes** ***n* = 104** **(5%)**	**No** ***n* = 1878** **(95%)**	**Yes** ***n* = 87** **(5%)**	**No** ***n* = 1769** **(95%)**	**Yes** *** n *= 160** **(9%)**	**No** *** n* = 1649** **(91%)**	**Yes** *** n* = 82** **(5%)**	**No** *** n* = 1439** **(95%)**
***trans*-Nonachlor**	-	-	-128 (-238, -19)^**^	-39 (-69, -10)^**^	-44 (-90, 2)^*^	-33 (-67, 1)^*^	-	-	-43 (-151, 64)	-36 (-65, -7)^**^	-	-	-	-
**PCB 118**	-	-	76 (-70, 221)	-16 (-43, 12)	-	-	-87 (-194, 19)	-7 (-35, 20)	-1 (-111, 108)	-12 (-39, 15)	-28 (-87, 31)	-8 (-37, 21)	-	-
**PCB 180**	-	-	-	-	-32 (-65, 1)^*^	-8 (-36, 20)	-	-	29 (-44, 102)	-21 (-42, 0)^*^	-	-	-	-
**Aroclor 1260**	-	-	-	-	-	-	-	-	-	-	-34 (-80, 13)	-17 (-42, 7)	-48 (-102, 6)*	-13 (-37, 11)
**Oxychlordane**	21 (-69, 111)	-30 (-60, -0)^**^	-124 (-236, -12)^**^	-28 (-58, 2)^*^	-	-	-	-	-	-	4 (-50, 57)	-35 (-68, -3)**	-93 (-177, -8)**	-19 (-49, 11)
**DDE**	-	-	-	-	-	-	26 (-41, 92)	-15 (-36, 5)	-	-	-	-	-	-

*n* indicates sample size;—indicates not selected by elastic net models

Each cell contains the difference in birth weight measured in grams and associated 95% confidence intervals

All models were stratified by hardship and adjusted for maternal education, maternal race and ethnicity, maternal age, maternal pre-pregnancy BMI, maternal smoking status, parity, infant sex and gestational age; Models assessing maternal hardship racially minoritized background were not adjusted for race and ethnicity; Models assessing maternal hardship low education or low income were not adjusted for maternal education; OC models additionally adjusted for total lipids

^**^p-value for association < 0.05

^*^p-value for association < 0.1

**Table 5:** The associations between exposure to metals (2-fold increase) and birth weight (grams) by maternal hardships among the MIREC study participants in Canada, 2008–2011

	**Low education**		**Low income**		**Lone parenthood**		**Racially minoritized background**		**Living alone**		**Student**	
	**Yes** ***n* = 160** **(9%)**	**No** ***n* = 1649** **(91%)**	**Yes** ***n* = 72** **(4%)**	**No** ***n* = 1698** **(96%)**	**Yes** ***n* = 87** **(5%)**	**No** ***n* = 1769** **(95%)**	**Yes** ***n* = 160** **(9%)**	**No** ***n* = 1649** **(91%)**	**Yes** ***n* = 72** **(4%)**	**No** ***n* = 1698** **(96%)**	**Yes** ***n* = 82** **(5%)**	**No** ***n* = 1439** **(95%)**
**Pb**	-100 (-215, 16)^*^	-34 (-64, -3)^**^	-	-	-	-	18 (-54, 89)	-50 (-82, -17)^**^	-	-	-	-
**Hg**	23 (-25, 71)	-9 (-24, 6)	-	-	-	-	-	-	-	-	-	-
**As**	-58 (-130, 13)	-5 (-25, 16)	-87 (-189, 15)^*^	-3 (-23, 18)	-100 (-201, 1)^*^	-5 (-25, 15)^*^	-	-	-	-	-	-
**Mn**	-	-	192 (-27, 410)^*^	-1 (-46, 44)	-242 (-454, -29)^**^	20 (-25, 64)	-	-	231 (-151, 612)	7 (-37, 50)	81 (-52, 213)	-0 (-47, 46)
**Cd**	-	-	-	-	-	-	-35 (-79, 9)	-8 (-31, 15)	-	-	-	-

*n* indicates sample size;—indicates not selected by elastic net models

Each cell contains the difference in birth weight measured in grams and associated 95% confidence intervals

All models were stratified by hardship and adjusted for maternal education, maternal race and ethnicity, maternal age, maternal pre-pregnancy BMI, maternal smoking status, parity, infant sex and gestational age; Models assessing maternal hardship racially minoritized background were not adjusted for maternal race and ethnicity; Models assessing maternal hardship low education or low income were not adjusted for maternal education; OC models additionally adjusted for total lipids

^**^p-value for association < 0.05

^*^p-value for association < 0.1

#### OCs

Among the OCs, we observed negative (e.g., *trans*-nonachlor) to no associations (e.g., PCB 118) with birth weight for females who did not report any of the hardships examined. For *trans*-nonachlor and Aroclor 1260, we observed larger than expected negative associations on mean birth weight among females with hardships (Fig. 1A and D). For example, each twofold increase in *trans*-nonachlor concentration was associated with a lower mean birth weight of -128 g (95% CI: -238, -19) among those with low income and -44 g (95% CI: -90, 2) among immigrants (Table 4). Meanwhile, infants born to higher income females or Canadian-born females had smaller birth weight reductions of 39 g (95% CI: -69, -10) and 33 g (95% CI: -67, 1), respectively. Similarly, we observed steeper negative slopes for those with increasing PCB 118 concentrations and belonging to a racially minoritized group (Fig. 1B-i), increasing PCB 118 concentrations and inadequate supplemental folic acid intake (Fig. 1B-ii), increasing PCB 180 concentrations and immigrant status (Fig. 1C-i), and increasing oxychlordane concentrations and low income (Fig. 1E-iii). Furthermore, students with increasing exposure to environmental chemicals also showed steeper curves and greater birth weight reduction, compared to non-students. However, the dose response curves crossed over indicating that students had initial gains in birth weight at low exposure compared to non-students but as exposure increased, greater reduction in birth weight was observed (Fig. 1D-ii and E-iv).

We observed a change in direction for the following associations: PCB 118 × low income (Fig. 1B-iii), PCB 180 × lone parenthood (Fig. 1B-iv), oxychlordane x low education (Fig. 1E-ii), and DDE x low supplemental folic acid intake (Fig. 1F). Among those who reported hardships, we saw a gain, on average, in birth weight of at least 21 g (95% CI: -69, 111) for infants born to females with increasing oxychlordane concentrations and low education to a maximum of 76 g (95% CI: -70, 221) for infants born to females with increasing PCB 118 concentrations and low income (Table 4). Conversely, among those who did not report a hardship, we saw reductions in mean birth weight of between 15 g (95: CI: -36, 5) for infants born to females with increasing DDE concentrations but had adequate supplemental folic acid intake and 30 g (95: CI: -60, -0) for infants born to females with increasing PCB 118 concentrations but had higher income (Table 4).

#### Metals

Among the metals, we also observed negative (e.g., Pb) to no associations (e.g., As) with birth weight for those who did not report hardships. For Pb exposure, we observed both a strengthening of association and change in direction when combined with hardships. Among infants born to females with increasing Pb concentrations and low educational status, we observed a larger reduction in mean birth weight ($\beta$ = -100 g; 95% CI: -215, 16) compared to those with higher educational status ($\beta$ = -34 g; 95% CI: -64, -3) (Fig. 2A-i and Table 5); while for infants born to females with increasing Pb concentrations and belonging to a racially minoritized group, we observed a positive association ($\beta$ = 18 g; 95% CI: -54, 89) and a gain of 68 g, on average compared to infants born to white females (Fig. 2 A-ii and Table 5). For As and Cd exposures, among those with hardships, we observed stronger associations (i.e., steeper slope) with birth weight compared to those who did not report hardships (Fig. 2B and E). Similarly to that of Pb, the associations between As and birth weight were stronger (steeper slope) among infants born to females with low educational status ($\beta$ = -58 g; 95% CI: -130, 13), low income ($\beta$ = -87 g; 95% CI: -189, 15), and lone parenthood ($\beta$ = -100 g; 95% CI: -201, 1) compared to their counterparts (Fig. 2B and Table 5). Furthermore, for Mn and Hg exposures, we observed positive associations among those who experienced hardships. For instance, infants born to females with low educational status, compared to those with higher educational status, had lower birth weight at low Mn or Hg concentration*.* As Mn or Hg level increased, the dose response curve trended upward leading to higher birth weights at high Mn or Hg concentrations (Fig. 2C and D). One exception to this pattern is lone parents with increasing Mn concentrations. A 242 g (95% CI: -454, -29) reduction in mean birth weight was observed among infants born to single females compared to females who were not single ($\beta$ = 20 g; 95% CI: -25, 64) (Fig. 2D-iii and Table 5).

### Supplemental analyses results

Low first trimester plasma total folate concentrations (i.e., < 5th percentile or < 51.5 nmol/L) (Table S4) was associated with a 124 g decrease in birth weight (95% CI: -223, -26) in the adjusted model (Table S5). From the Cramer’s V correlation coefficient plot (Figure S4), we observed low correlations (0 to 0.2) between low plasma total folate and other maternal hardship variables.

When we included low plasma total folate (instead of low supplemental folic acid intake) in our elastic net model, the selections of variables by elastic net for both the OC and the metal models were similar except that in the metal models, Hg and immigrant status were additionally selected. In the adjusted models, we observed that every doubling of Hg concentration corresponded to a 6 g (95% CI: -21, 8) decrease in birth weight and infants born to immigrants faced a 21 g (95% CI: -78, 35) reduction in birth weight compared to those born to non-immigrants (Table S5). Elastic net also identified three interactions associated with Hg as important: Hg x low income, Hg x low plasma total folate and Hg x chronic diseases (Table S6). The interaction plot of Hg x chronic diseases showed a stronger association (steeper slope) indicating greater detriments in birth weight at higher Hg concentration for those who reported having one or more chronic diseases compared to those who did not report any. Meanwhile, the interaction plots of Hg x low income and Hg x low plasma total folate showed positive associations where the dose response curves for those who reported hardship had positive slopes (Figure S5). Additionally, we found that adjustment for GA as a covariate attenuated the relationships between exposure to chemical concentrations and birth weight (Table S5).

Our cumulative hardship analysis found that the proportions of participants who reported no hardship, one hardship, and two or more hardships were 43%, 33%, and 24% respectively (Table S1). We observed a negative relationship where a higher number of hardships corresponded to lower mean birth weight (Table S1). In the adjusted model, the presence of one hardship or two or more hardships corresponded to lower birth weight of 58 g (95% CI: -106, -11) and 93 g (95% CI: -148, -39), respectively, compared to no hardship (Table S7).

## Discussion

Our findings suggest that maternal hardships that co-occur with gestational exposure to some OCs and metals, may interact and produce greater detrimental effects on fetal growth than either exposure alone. For instance, *trans*-nonachlor and oxychlordane were more strongly associated with decreased infant birth weight among females with lower income compared to those with higher income. This finding is not unexpected as it has long been recognized that females with lower income have disproportionately higher exposure for environmental stressors as well as higher risk for impaired fetal growth [52]. For instance, Borders et al. (2007) found, among their population of 1,363 pregnant American females with low income, that maternal social hardships and low birth weight were strongly related. They also reported that pregnant females with lower income are more likely to face food insecurity and consume inadequate supplemental folic acid compared to those with higher income [53]. This relationship among poverty, food insecurity, and folic acid supplementation is well supported [30, 31, 54]. In our study, 104 (5%) participants had low supplemental folic acid intake; only 10 (1%) experienced both low income and low supplemental folic acid intake. Among those infants born to females with increasing PCB 118 concentrations and low supplemental folic acid intake, we observed that every twofold increase in PCB 118 concentration was associated with an 87 g reduction in birth weight among females with low supplemental folic acid intake, compared to a 7 g reduction among females with the recommended supplemental folic acid intake level.

Beside maternal income, maternal education also had a strong and positive association with birth weight [55]. In our study, Pb x low education was one of the selected interaction terms by elastic net. Pb exposure is unequally distributed across populations where higher exposure to Pb is typically found in communities of lower socioeconomic status and among individuals with less access to resources including financial, educational, social, and health [56]. Furthermore, both Pb exposure and low maternal education can independently contribute to lower birth weight [6, 7, 55, 57, 58]. In combination, we found that Pb was associated with a greater reduction in birth weight among females with low educational status ($\beta$ = -100 g; 95% CI: -215, 16) compared to those who had higher education ($\beta$ = -34 g; 95% CI: -64, -3) (Table 5).

Another hardship that has been known to affect birth weight is immigration status. When we considered the females’ immigration status along with their exposure to OCs (i.e., *trans*-nonachlor and PCB 180), immigrants showed a lower mean birth weight compared with Canadian-born participants (Fig. 2A,C and Table 4). This birth weight reduction is supported by an increasing number of studies that found that immigration status affects fetal growth [59] and that the birth weights of babies born to immigrants are generally lower than those of babies born to Canadian-born females [60, 61]. For example, South Asian born females tend to give birth to smaller babies than non-migrant females [62]. However, in a systematic review where Gagnon et al. (2009) explored whether immigrants have poorer infant outcomes compared to Canadian-born females, the authors found that being an immigrant was not a consistent marker for poorer infant outcomes [60]. Nevertheless, among our MIREC population, immigrants had lower mean birth weights and when considering the effects of environmental chemical exposures, they experienced a slight increase in vulnerability compared with Canadian-born females. Interestingly, we also observed a greater reduction in birth weight among infants born to students with increasing concentrations of OCs (i.e., Aroclor 1260 and PCB 180), compared to those of non-students. There is a dearth of studies on environmental chemical exposures in the context of pregnant students. Available studies typically examined behaviours in reducing exposure [63], knowledge and awareness of exposures to environmental chemicals [64], or exposure assessment on campus or in laboratories [65]. Future work, therefore, should include pregnant students as the intersection of their experiences and exposure status may generate differential impacts on their pregnancy outcomes.

The stronger relationships we observed for females with both higher environmental chemical concentrations and a maternal hardship is consistent with Knudson’s “two-hit” hypothesis [66] where two distinct gestational stressors (e.g., gestational exposures to environmental chemical and maternal hardship) showed different associations with birth weight when combined compared to either “hit” alone. To further explore the impact of cumulative effects, we summed the number of hardships each participant faced and found that as the number of hardships increased, the mean birth weight decreased (Table S1). Specifically, the presence of one hardship was associated with a 59 g (95% CI: -105, -12) lower birth weight while the presence of two or more hardships was associated with an 87 g (95% CI: -139, -34) lower birth weight, compared to no hardship (Table S7). While the findings of the present study support the hypothesis that exposure to two stressors can have different effects compared to exposure to a single stressor, these findings may depend on the nature of the stressor(s). For instance, infants born to MIREC participants with increasing Mn or Hg concentrations and reported maternal hardship had higher birth weights compared to those born to participants who did not report hardship.

Studies had shown that Mn exhibits a nonlinear inverse U-shaped relationship with birth weight where lower birth weight was observed at both low and high concentrations of blood Mn level [67, 68]. Despite that, most studies, including the present one, assumed linear functions. In our previous study, using a flexible Bayesian Kernel Machine Regression method that can accommodate non-linearity, we found that Mn showed a positive and linear relationship with infant birth weight among MIREC participants [7]. It is possible that we have captured the ascending segment of the inverted U-shaped Mn-birth weight relationship, which showed an association with higher birth weight in our adjusted regression analysis. As a result, when examined by maternal hardship status, we found evidence that infants born to MIREC participants with both higher Mn concentration and low income had a higher birth weight, while those with no reported hardship showed no association with birth weight. However, the pattern doesn't hold true for lone parenthood as infants born to single females experienced approximately 200 g greater birth weight reduction compared to those born to married parents. The limited research on lone parenthood (or single mother families) and environmental chemical exposures has focused on exposure to air pollutants. Two US-based environmental inequality studies reported that single-mother families are more likely to live in neighborhoods with higher pollution compared to other family types (e.g., married or single father families) [69, 70]. Another US air pollution study found that living in a poor neighbourhood may increase the risk of exposure to OCs and metals in the air [71]. We are not aware of any study that has examined the combined effects of lone parenthood and Mn exposure on pregnancy outcomes. Research that assesses the relationship between wider ranges in Mn concentrations and fetal growth is highly recommended to consider the shape of the Mn-birth weight function as well as the impact of lone parenthood.

Like Mn, total Hg also showed a positive relationship with birth weight among those with maternal hardships. Although Hg is a toxic chemical that can freely cross the placenta and potentially disrupt a range of important pregnancy processes [72], in a recent systematic review, Hg was reported to have a minimal to null association with birth weight [73]. However, one particular study reported that the lowest tertile hair Hg concentration was associated with a higher risk of having infants with low birth weight (adjusted odds ratio (aOR) = 7.2; 95% CI: 1.5, 35.6) compared to those with higher Hg concentration (aOR = 0.52; 95% CI: 0.17, 1.55) [74]. As Hg exposure generally results from maternal fish consumption, the authors hypothesized that the consumption of contaminated fish with high levels of Hg may have coincided with an increased intake of selenium (Se), an essential element found in fish. Se, in this case, may have moderated the toxic effects of Hg [75, 76] and thus resulted in a decreased OR for low-birth-weight infants among those with higher Hg exposure. Other studies have also reported that Hg levels have a positive association with socioeconomic status among females of childbearing age [77, 78]. Specifically, pregnant participants with low income and low education consumed more fish per week compared to pregnant participants with higher income and higher educational status, but they were found to have lower blood Hg levels [78]. This is because the type of fish consumed by different demographic groups can also affect Hg exposure levels [78]. Therefore, future studies should include Se, the types of fish consumed, and if possible, the whole diet when assessing the potential toxic effects of Hg.

Among the MIREC study participants, we observed that maternal hardships can modify the strength of the relationships (i.e., steeper negative slope and lower birth weight) as well as the direction of the relationships (i.e., positive slope and higher birth weight) between biomarkers of exposures to OCs and metals during pregnancy and infant birth weight. To our knowledge, this study is the largest single cohort study conducted on the modifying effect of maternal hardship on the relationship between gestational environmental chemical concentrations and birth weight. With the benefits of a large sample size and the use of elastic net regularization technique, we had higher statistical power to detect associations and identify key variables. However, our findings should be interpreted with caution due to the following limitations. First, we used multivariable linear regression to assess the independent effects of each maternal hardship and environmental chemical on birth weight and did not account for non-linear relationships. Second, we performed post-selection inference by first using elastic net for variable selection, then using OLS to derive statistical inference from the best-fitting model. A limitation of variable selection methods such as elastic net is their instability where any change in observations may change the model selected [79]. As OLS assumes that we have obtained a perfect model, which may not be the case, the resulting 95% CIs may be inaccurate and too narrow. Accounting for the effects of variable selection is a challenging task and work is underway to develop tools for selective inference so that we can properly assess the strength of the relationships [79]. Third, we used Lubin’s single imputation approach for measurements below the LOD and the standard errors may be biased. However, as the majority of our environmental chemicals were detected in over 90% of the participants, the impact of bias, if any exists, should be minimal [22]. Fourth, maternal hardships were based on a positive–negative dichotomy (i.e., yes–no hardship), which produced easy to interpret findings but essentially weighted all hardships equally. This assumption may have overlooked important and differential aspects of the potential effect of each hardship on the outcome*.* Furthermore, low maternal hardship proportions of 2 to 25% and missing maternal hardship values may bias the effect estimates, reduce our study power to detect a statistically significant association, and induce spurious interactions. Regardless, our use of dichotomized hardships served as an important starting point for the conceptualization of maternal hardships and highlighted the importance for future research to include parental hardships when examining the effects of environmental chemical exposure. Additionally, our use of objective maternal hardships may not fully represent a woman’s experience and well-being [80]. Obtaining qualitative data and additional quantitative data such as food security, stressful life events, and resilience will enhance our understanding of maternal hardships and enrich our current findings. Fifth, we examined only OCs and metals and did not consider co-exposures among the environmental chemicals or hardships. Therefore, we may have missed other environmental chemicals-birth weight associations modified by maternal hardships or any potential 2-way interactions between environmental chemicals or between hardships. Sixth, blood may not be an adequate biomarker of exposure to all metals as each medium may represent a different window of exposure [81]. For example, blood Cd, As and Hg reflect recent exposures, while urinary Cd, hair or nail As and Hg may better assess chronic exposures [81–83]. Lastly, MIREC is not a representative sample of all Canadian females because it is not population-based [16]. As a result, the generalizability of our results may be limited.

## Conclusions

While it is widely agreed that fetal growth is determined by multiple factors encompassing genetic, environmental, social and maternal factors, little is known about how these factors work together to maintain the persistent disparities in abnormal fetal growth. We present supporting evidence that maternal hardships may modify the relationship between gestational environmental chemical exposures and infant birth weight and that two “hits” of stressors have different effects compared to a single “hit”. Future research should examine the mixture or cumulative effects of environmental chemicals and non-chemical stressors such as maternal hardships to further advance our knowledge of the mechanisms of impaired fetal growth. Greater knowledge can address the adverse fetal growth outcomes where vulnerable populations are at increased risk for impaired fetal growth. These hardships or social factors that co-occur with gestational environmental chemical exposure can become a source of inequality and have downstream effects on infant health [13].

### Supplementary Information

Supplementary Material 1.

## Data Availability

Due to data privacy issues, we are not able to make these data publicly available. Individuals may apply to access the data through the MIREC Biobank (www.mirec-canada.ca/en/research).

## Abbreviations

aOR: Adjusted odds ratio
As: Arsenic
Cd: Cadmium
CI: Confidence interval
DDE: Dichlorodiphenyldichloroethylene
GA: Gestational age
g: Grams
Hg: Mercury
INSPQ: Institut national de santé publique du Québec
LOD: Limit of detection
MIREC: Maternal-Infant Research on Environmental Chemicals
Mn: Manganese
NTD: Neural tube defects
OC: Organochlorines
OLS: Ordinary least squares
OR: Odds ratio
Pb: Lead
PCB: Polychlorinated biphenyl
PROTECT: Puerto Rico Testsite for Exploring Contamination Threats
SD: Standard deviation
Se: Selenium
