# Correction: Gestational exposure to organochlorine compounds and metals and infant birth weight: effect modification by maternal hardships

**DOI:** 10.1186/s12940-024-01109-8

**Published:** 2024-08-30

**Authors:** Janice M. Y. Hu, Tye E. Arbuckle, Patricia A. Janssen, Bruce P. Lanphear, Joshua D. Alampi, Joseph M. Braun, Amanda J. MacFarlane, Aimin Chen, Lawrence C. McCandless

**Affiliations:** 1 Environmental Health Science and Research Bureau, Healthy Environments and Consumer Safety Branch, Health Canada, 101 Tunney’s Pasture Driveway, Ottawa, ON K1A 0K9 Canada; 2 Faculty of Health Sciences, Simon Fraser University, Burnaby, BC V5A 1S6 Canada; 3 School of Population and Public Health, University of British Columbia, Vancouver, BC V6T 1Z4 Canada; 4 Department of Epidemiology, Brown University, Providence, RI USA; 5 Texas A&M Agriculture, Food and Nutrition Evidence Center, Fort Worth, TX USA; 6 Department of Biostatistics, Epidemiology and Informatics, University of Pennsylvania Perelman School of Medicine, Philadelphia, PA USA

**Correction: Environ Health 23, 60 (2024)**

10.1186/s12940-024-01095-x

Following publication of the original article [1], the author reported that the copyrightline of their article should be: ©His Majesty the King in Right of Canada, as represented by the Minister of Health, 2024.
