# Supplementary material for: Gestational exposure to organochlorine compounds and metals and infant birth weight: effect modification by maternal hardships
Source: Environ Health. 2024 Jul 1;23:60. doi: 10.1186/s12940-024-01095-x (PMC11218229; doi:10.1186/s12940-024-01095-x)
Supplement: Supplementary file 1 — Supplementary Material 1. [file 12940_2024_1095_MOESM1_ESM.docx]

**Manuscript Title:**Gestational exposure to organochlorine compounds and metals with infant birth weight: Effect modification by maternal hardships

## **Authors:** Janice M.Y. Hu^a^, Tye E. Arbuckle^b^, Patricia A. Janssen^c^, Bruce P. Lanphear^a^, Joseph M. Braun^d^, Amanda J. MacFarlane^e^, Aimin Chen^f^, Joshua D. Alampi^a^, Lawrence C. McCandless^a^

**Affiliations:**^a^ Faculty of Health Sciences, Simon Fraser University, Burnaby, BC V5A 1S6, Canada
^b^ Population Studies Division, Environmental Health Science and Research Bureau, Healthy Environments and Consumer Safety Branch, Health Canada, Ottawa, ON, Canada
^c^ School of Population and Public Health, University of British Columbia, Vancouver, BC V6T 1Z4, Canada

^d^ Department of Epidemiology, Brown University, Providence, RI, USA

^e^ Texas A&M Agriculture, Food, and Nutrition Evidence Center, Fort Worth, TX, USA

^f^ Department of Biostatistics, Epidemiology and Informatics, University of Pennsylvania Perelman School of Medicine, Philadelphia, PA, USA

**Corresponding author:**

Janice Hu, Faculty of Health Sciences, Simon Fraser University, 8888 University Drive,

Burnaby, BC V5A 1S6, Canada; mungh@sfu.ca

**Supplemental Tables and Figures:**

**Figure S1.** Study sample flowchart 3

**Figure S2.** Directed Acyclic Graph for the relation among potential environmental chemical concentrations during pregnancy, infant birth weight, maternal hardships and various demographic characteristics 4

**Figure S3.** Cramer’s V correlation coefficients examining the strength of the relationships between hardships 5

**Table S1.** Participant cumulative hardships and mean birth weight (grams) among MIREC study participants in Canada, 2008-2011 5

**Table S2.** Frequency and percentage of joint maternal hardships 6

**Table S3.** Distributions of environmental chemicals measured during the 1st trimester for MIREC study participants in Canada, 2008-2011. 7

**Table S4.** Participant plasma total folate status and mean birth weight (grams) among MIREC study participants in Canada, 2008-2011. 7

**Table S5.** Differences in mean birth weight (grams) and 95% CI associated with exposure to plasma folate inadequacy among the MIREC study participants in Canada, 2008-2011. 7

**Figure S4.** Cramer’s V correlation coefficients examining the strength of the relationships between hardships (including low plasma total folate) 8

**Figure S5.** Interaction plot showing the differences in mean birth weight (grams) and 95% confidence intervals (shaded bands) associated with exposures to blood mercury concentrations during the first trimester for participants with and without specific hardships 8

**Table S6.** The associations between exposures to environmental chemicals (2-fold increase) and birth weight (grams) by hardship status (including low plasma total folate), mutually adjusted for one another and covariates, among the MIREC study participants in Canada, 2008-2011 9

**Table S7.** Difference in mean birth weight (grams) and 95% confidence interval associated with exposure to the number of hardships among the MIREC study participants in Canada, 2008-2011. 12

# **Appendix:**

# **Appendix A.** Laboratory analysis 13

# **Appendix B.** Descriptive statistics (complete cases vs. incomplete cases) 14


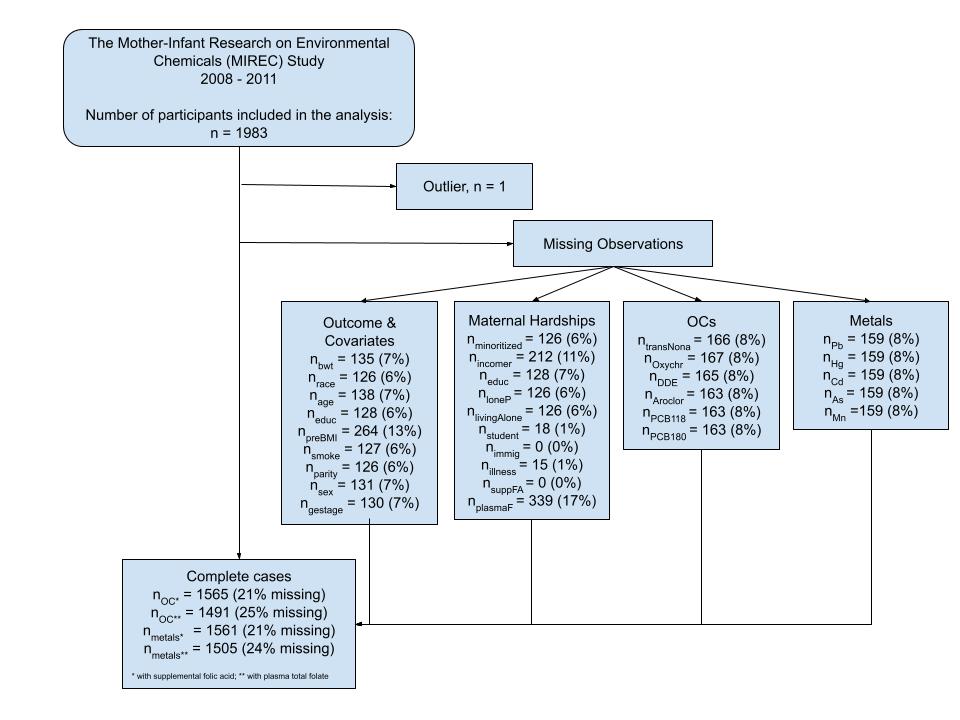
**Figure S1.** Study sample flowchart.


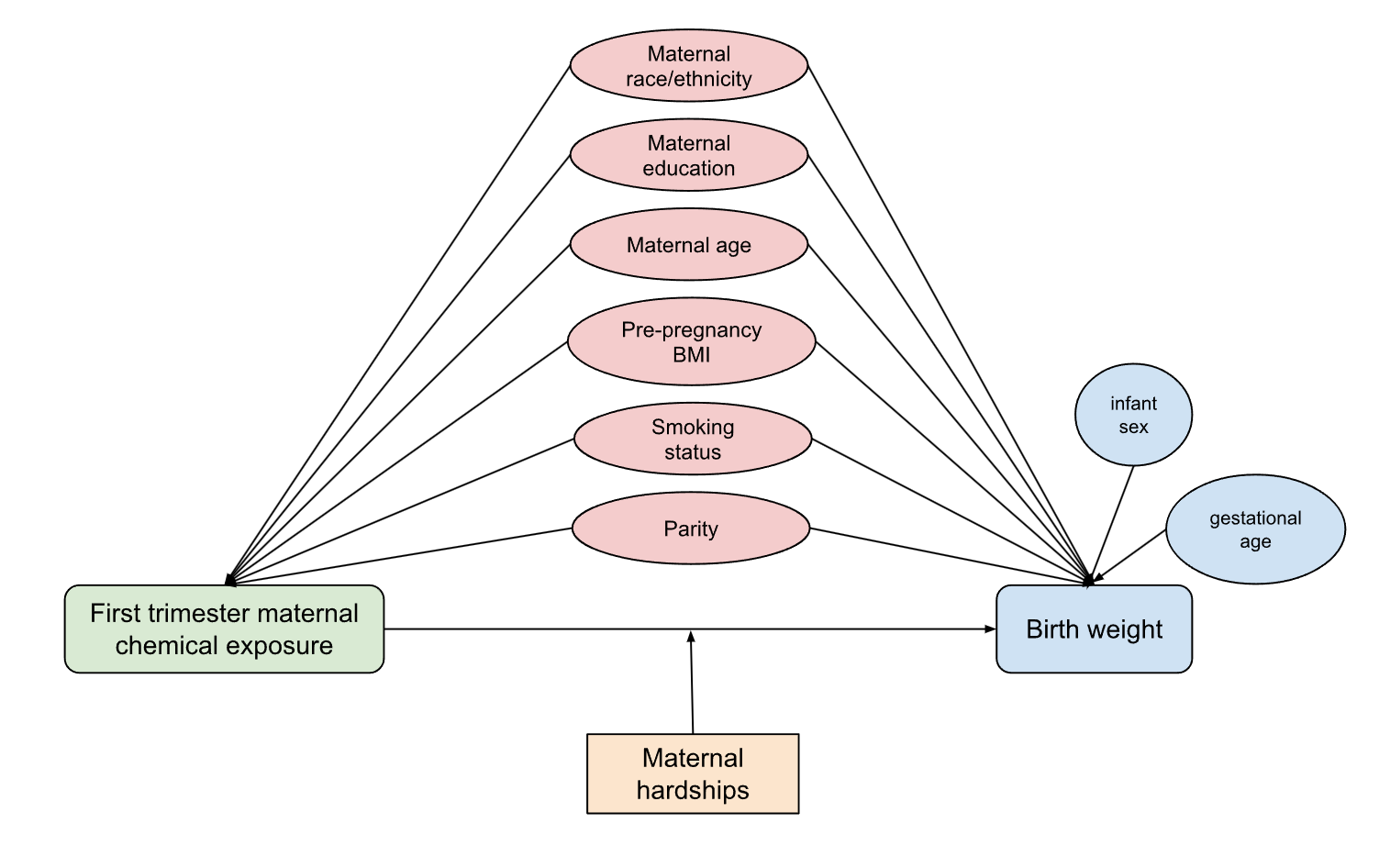


**Figure S2.** Directed Acyclic Graph (DAG) for the relation among gestational exposure to environmental chemical concentrations, infant birth weight, maternal hardships and various covariates. The organochlorine models also adjusted for total lipids. Interaction model assessing hardship race and ethnicity did not adjust for race. Interaction models assessing hardships low education and low income did not adjust for maternal education. Gestational age may be a mediator.


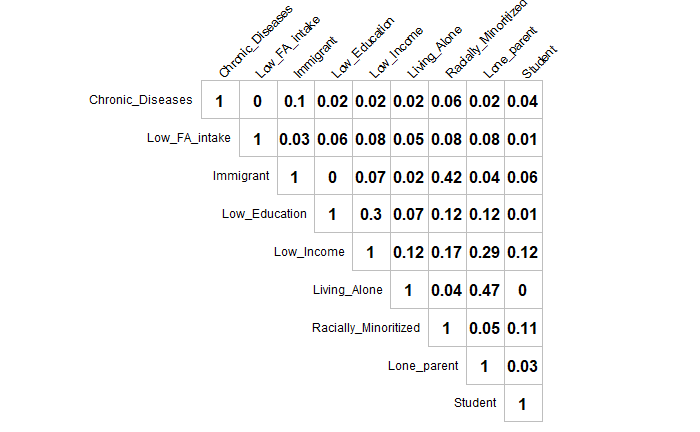


**Figure S3.** Cramer’s V correlation coefficients examining the strength of the relationships between hardships.

| **Table S1.** Participant cumulative hardships and mean birth weight (grams) among MIREC study participants in Canada, 2008-2011. **A.** Cumulative hardship with low supplemental folic acid intake. **B.** Cumulative hardship with low plasma total folate. | | |
| --- | --- | --- |
|  | **n (%)** | **Birth weight (g)**  **Mean (SD)** |
| Total | 1982 (100) | 3452 (532) |
| **A.** Cumulative hardship (with low supplemental folic acid intake) | | |
| 0 | 751 (43) | 3529 (489) |
| 1 | 569 (33) | 3419 (559) |
| 2+ | 424 (24) | 3369 (540) |
| **B.** Cumulative hardship (with low plasma total folate) | | |
| 0 | 707 (43) | 3521 (496) |
| 1 | 538 (33) | 3425 (540) |
| 2+ | 398 (24) | 3373 (542) |
| *Cumulative hardship is a sum of the number of hardships each participant faces. | | |

| **Table S2.** Frequency and percentage of joint maternal hardships. | | | | | | | | | |
| --- | --- | --- | --- | --- | --- | --- | --- | --- | --- |
|  | **Racially minoritized** | **Low education** | **Low income** | **Living alone** | **Immigrant** | **Low supplemental folic acid intake** | **Low plasma total folate** | **Lone parenthood** | **Current student** |
| **n (%)** | **339 (18)** | **160 (9)** | **72 (4)** | **36 (2)** | **370 (19)** | **104 (5)** | **92 (5)** | **87 (5)** | **201 (10)** |
| **Racially minoritized** | - | 54 (3%) | 35 (2%) | 11 (1%) | 183 (10%) | 31 (2%) | 23 (1%) | 23 (1%) | 57 (3%) |
| **Low education** | 54 (3%) | - | 34 (2%) | 8 (0%) | 30 (2%) | 15 (1%) | 22 (1%) | 21 (1%) | 14 (1%) |
| **Low income** | 35 (2%) | 34 (2%) | - | 7 (0%) | 23 (1%) | 10 (1%) | 15 (1%) | 24 (1%) | 20 (1%) |
| **Living alone** | 11 (1%) | 8 (0%) | 7 (0%) | - | 9 (0%) | 5 (0%) | 4 (0%) | 27 (1%) | 4 (0%) |
| **Immigrant** | 183 (10%) | 30 (2%) | 23 (1%) | 9 (0%) | - | 24 (1%) | 16 (1%) | 11 (1%) | 51 (3%) |
| **Low supplemental folic acid intake** | 19 (1%) | 6 (0%) | 4 (0%) | 1 (0%) | 15 (1%) | - | 5 (0%) | 5 (0%) | 8 (1%) |
| **Low plasma total folate** | 23 (1%) | 22 (1%) | 15 (1%) | 4 (0%) | 16 (1%) | 26 (1%) | - | 5 (0%) | 8 (1%) |
| **Lone parenthood** | 23 (1%) | 21 (1%) | 24 (1%) | 27 (1%) | 11 (1%) | 12 (0%) | 5 (0%) | - | 13 (1%) |
| **Current student** | 57 (3%) | 14 (1%) | 20 (1%) | 4 (0%) | 51 (3%) | 12 (1%) | 8 (1%) | 13 (1%) | - |
| n (%) indicates sample size and percentage | | | | | | | | | |

| **Table S3.**  Distributions of environmental chemicals measured during the 1st trimester for MIREC study participants from 2008 to 2011 across 10 major cities in Canada. | | | | | | | | |
| --- | --- | --- | --- | --- | --- | --- | --- | --- |
| Metabolites | %>LOD | LOD | GM^1^ | Min | 25th | 50th | 75th | Max |
| **Organochlorine compounds (ng/g lipids)** | | | | | | | | |
| PCB 118 | 73.6 | 0.01 | 2.5 | <LOD | 1.7 | 2.3 | 3.4 | 37.9 |
| PCB 180 | 92.7 | 0.01 | 4.9 | <LOD | 2.9 | 4.8 | 7.8 | 183.3 |
| Aroclor 1260 | 97.3 | 0.10 | 60.7 | <LOD | 37.0 | 57.9 | 90.9 | 1183.3 |
| DDE | 99.0 | 0.09 | 56.4 | <LOD | 33.8 | 49.1 | 77.9 | 530.6 |
| Oxychlordane | 92.3 | 0.005 | 2.0 | <LOD | 1.4 | 2.1 | 2.9 | 17.5 |
| *trans*-  Nonachlor | 84.3 | 0.01 | 3.0 | <LOD | 2.0 | 2.9 | 4.2 | 34.3 |
| **Metals (ug/L)** | | | | | | | | |
| Arsenic | 92.5 | 0.22 | 0.8 | <LOD | 0.2 | 0.7 | 1.1 | 34.5 |
| Cadmium | 97.4 | 0.04 | 0.2 | <LOD | 0.5 | 0.2 | 0.3 | 5.1 |
| Mercury | 90.5 | 0.12 | 0.6 | <LOD | 0.1 | 0.7 | 1.4 | 10.0 |
| Manganese | 100.0 | 0.55 | 8.8 | 2.0 | 0.3 | 8.8 | 11.0 | 29.1 |
| Lead | 100.0 | 0.10 | 6.2 | 1.6 | 7.1 | 6.0 | 8.5 | 41.4 |
| ^1^GM = Geometric Mean | | | | | | | | |

| **Table S4.** Participant plasma total folate status and mean birth weight (grams) among MIREC study participants in Canada, 2008-2011. | | |
| --- | --- | --- |
|  | **n (%)** | **Birth weight (g)**  **Mean (SD)** |
| Total | 1982 (100) | 3452 (532) |
| **Low plasma total folate (<51.5 nmol/L)** | | |
| Yes | 92 (5) | 3385 (494) |
| No | 1753 (95) | 3454 (531) |

| **Table S5.** Differences in mean birth weight (grams) and 95% CI associated with exposure to plasma folate inadequacy among the MIREC study participants in Canada, 2008-2011 | | | |
| --- | --- | --- | --- |
|  | Unadjusted | Adjusted for  covariates* | Adjusted for  covariates** |
| **Metal** | | | |
| Hg | -8 (-24, 9) | -6 (-21, 8) | 3 (-15, 22) |
| **Maternal Hardship** | | | |
| Low plasma total folate  (<51.5 nmol/L) | -69 (-182, 44) | -124 (-223, -26) | -77 (-202, 48) |
| Immigrant | -90 (-152, -29) | -21 (-78, 35) | -14 (-85, 58) |
| * Model was adjusted for gestational age (GA), maternal race, maternal education, maternal age, maternal BMI, smoking, parity, and baby sex.  ** Adjusted for all covariates listed above except for GA. | | | |


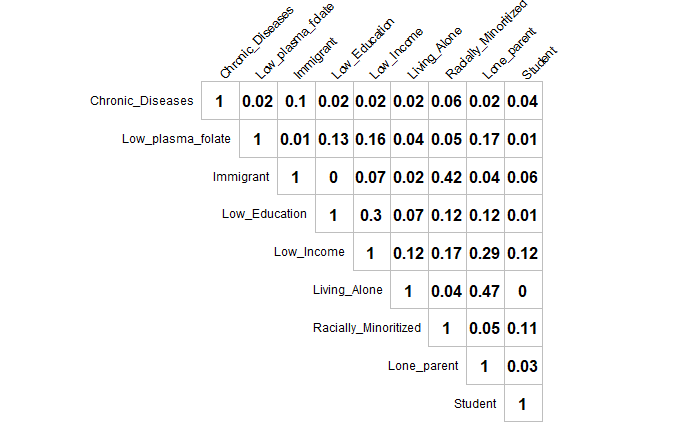


**Figure S4.** Cramer’s V correlation coefficients examining the strength of the relationships between hardships (including low plasma total folate).


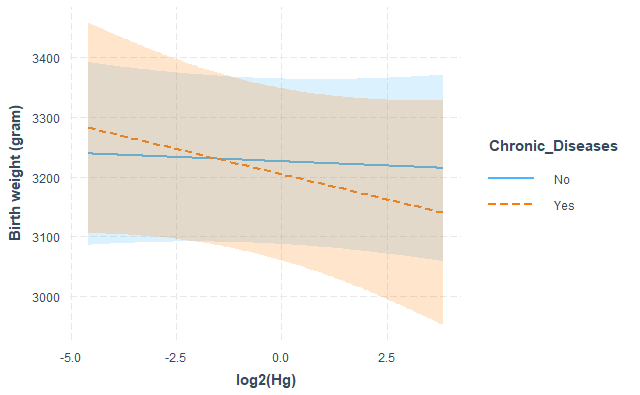

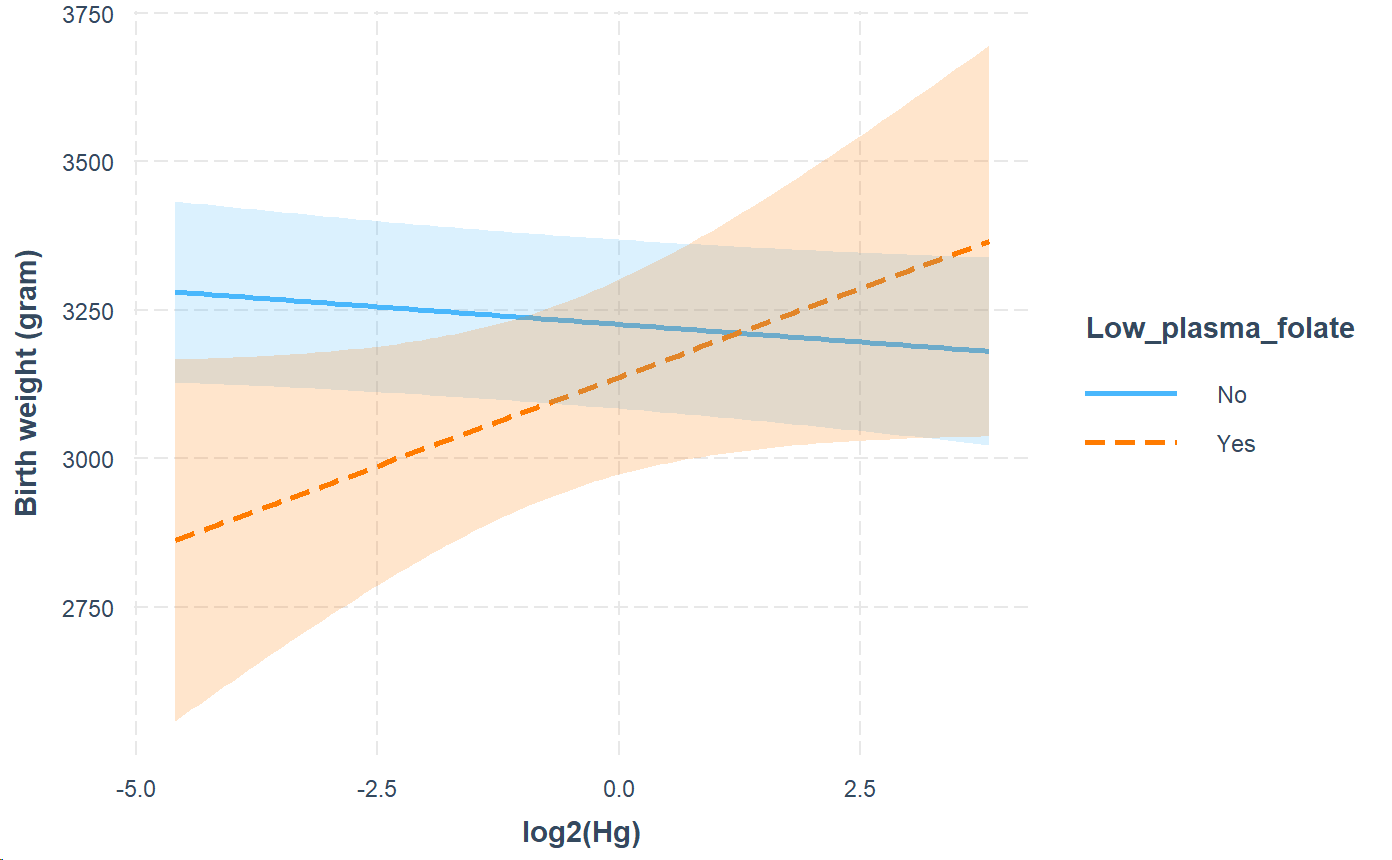

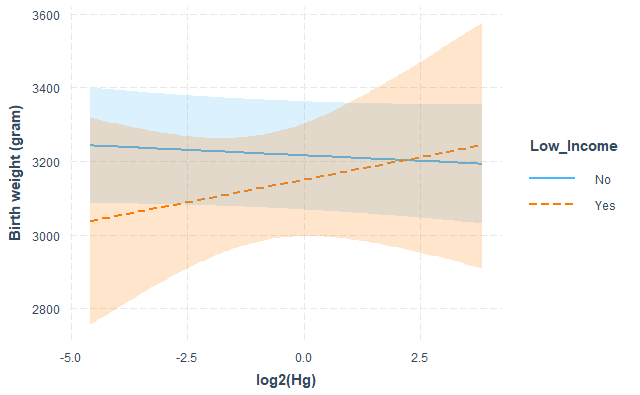


**Figure S5.** Interaction plots as selected by elastic net showing the differences in mean birth weight (grams) and 95% confidence intervals (shaded bands) associated with exposures to mercury during the first trimester for participants with and without specific hardships.

| **Table S6.** The associations between exposures to environmental chemicals (2-fold increase) and birth weight (grams) by hardship status (including low plasma total folate) among the MIREC study participants in Canada, 2008-2011. | | | | | | | | | | |
| --- | --- | --- | --- | --- | --- | --- | --- | --- | --- | --- |
|  | **Low education** | | **Low income** | | **Immigrant** | | **Low plasma total folate^1^** | | **Lone parenthood** | |
|  | **Yes**  **n = 160**  **(9%)** | **No**  **n = 1649**  **(91%)** | **Yes**  **n = 72**  **(4%)** | **No**  **n = 1698**  **(96%)** | **Yes**  **n = 370 (19%)** | **No**  **n = 1612**  **(81%)** | **Yes**  **n = 92**  **(5%)** | **No**  **n = 1753**  **(95%)** | **Yes**  **n = 87**  **(5%)** | **No**  **n = 1769**  **(95%)** |
| ***trans*-**  **nonachlor** | - | - | -128 (-238, -19)** | -39 (-69, -10)** | - | - | - | - | -43 (-151, 64) | -36 (-65, -7)** |
| **PCB 118** | - | - | 76 (-70, 221) | -16 (-43, 12) | - | - | - | - | - | - |
| **PCB 180** | - | - | - | - | -32 (-65, 1)* | -8 (-36, 20) | - | - | - | - |
| **Aroclor 1260** | - | - | - | - | - | - | - | - | - | - |
| **DDE** | - | - | - | - | - | - | - | - | - | - |
| **Oxychlordane** | - | - | - | - | - | - | - | - | - | - |
| **Pb** | -100 (-215, 16)* | -34 (-64, -3)** | - | - | - | - | - | - | - | - |
| **Hg** | 23 (-25, 71) | -9 (-24, 6) | 24 (-40, 89) | -6 (-21, 9) | - | - | 60 (-5, 125)* | -12 (-27, 3) | - | - |
| **As** | -58 (-130, 13) | -5 (-25, 16) | -87 (-189, 15)* | -3 (-23, 18) | - | - | - | - | -100 (-201, 1)* | -5 (-25, 15) |
| **Mn** | - | - | 192 (-27, 410)* | -1 (-46, 44) | - | - | - | - | -242 (-454, -29)** | 20 (-25, 64) |
| **Cd** | - | - | - | - | - | - | -67 (-141, 6)* | -9 (-31, 14) | - | - |
| n indicates sample size; - indicates not selected by elastic net models.  Each cell contains the difference in birth weight measured in grams and 95% confidence interval.  All models were adjusted for maternal education, maternal race and ethnicity, maternal age, maternal pre-pregnancy BMI, maternal smoking status, parity, infant sex and gestational age. Models including maternal hardships low education and low income were not adjusted for education; OC models additionally adjusted for lipid dilution.  ** p-value for association < 0.05; * p-value for association < 0.1.  ^1^ equivalent to <51.5 nmol/L | | | | | | | | | | |

| **Table S6 (con’t).** The associations between exposure to environmental chemicals (2-fold increase) and birth weight (grams) by maternal hardships (including low plasma total folate) among the MIREC study participants in Canada, 2008-2011. | | | | | | | | |
| --- | --- | --- | --- | --- | --- | --- | --- | --- |
|  | **Racially Minoritized** | | **Living alone** | | **Student** | | **Chronic Illness(es)** | |
|  | **Yes**  **n = 160**  **(9%)** | **No**  **n = 1649**  **(91%)** | **Yes**  **n = 72**  **(4%)** | **No**  **n = 1698**  **(96%)** | **Yes**  **n = 82**  **(5%)** | **No**  **n = 1439**  **(95%)** | **Yes**  **n = 491**  **(25%)** | **No**  **n = 1476**  **(75%)** |
| ***trans*-**  **Nonachlor** | - | - | - | - | - | - | - | - |
| **PCB118** | - | - | - | - | - | - | - | - |
| **PCB180** | - | - | - | - | - | - | - | - |
| **Aroclor 1260** | -34 (-80, 13) | -17 (-42, 7) | - | - | - | - | - | - |
| **DDE** | - | - | - | - | - | - | - | - |
| **Oxychlordane** | - | - | - | - | -93 (-177, -8)** | -19 (-49, 11) | - | - |
| **Pb** | 18 (-54, 89) | -50 (-82, -17)** | - | - | - | - | - | - |
| **Hg** | - | - | - | - | - | - | -17 (-44, 10) | -3 (-19, 14) |
| **As** | 22 (-22, 66) | -16 (-39, 6) | - | - | - | - | - | - |
| **Mn** | - | - | 231 (-151, 612) | 7 (-37, 50) | 81 (-52, 213) | -0 (-47, 46) | - | - |
| **Cd** | -35 (-79, 9) | -8 (-31, 15) | - | - | - | - | - | - |
| n indicates sample size; - indicates not selected by elastic net models.  Each cell contains the difference in birth weight measured in grams and associated 95% confidence intervals.  All models were adjusted for maternal education, maternal race and ethnicity, maternal age, maternal pre-pregnancy BMI, maternal smoking status, parity, infant sex and gestational age; Models assessing maternal hardship low education or low income were not adjusted for maternal education; OC models additionally adjusted for total lipids.  ** p-value for association < 0.05; * p-value for association < 0.1 | | | | | | | | |

| **Table S7.** Difference in mean birth weight (grams) and 95% confidence interval associated with exposure to the number of hardships among the MIREC study participants in Canada, 2008-2011. | | |
| --- | --- | --- |
|  | Unadjusted | Adjusted for  covariates* |
| **Maternal Hardships** | | |
| Cumulative hardship (with low supplemental folic acid intake)  0  1  2+ | Ref  -110 (-167, -52)  -160 (-223, -97) | Ref  -59 (-105, -12)  -87 (-139, -34) |
| Cumulative hardship (with low plasma total folate)  0  1  2+ | Ref  -96 (-155, -37)  -148 (-212, -84) | Ref  -58 (-106, -11)  -93 (-148, -39) |
| *Adjusted for gestational age (GA), maternal age, maternal pre-pregnancy BMI, maternal smoking, parity, and baby sex. | | |

**Appendix A.** Laboratory analysis

All chemical analyses of first trimester blood samples were performed at the Toxicology Laboratory of the Institut national de santé publique du Québec (INSPQ), a laboratory accredited by the Standards Council of Canada.

Standardized operating procedures were developed to measure the chemicals and their metabolites. The analysis methods were previously described in detail ([Arbuckle et al., 2014](https://www.sciencedirect.com/science/article/pii/S0160412015001373#bb0025), [Langlois et al., 2014](https://www.sciencedirect.com/science/article/pii/S0160412015001373#bb0225)). Briefly, metals were measured in whole blood using inductively coupled plasma mass spectrometry. Concentrations were reported in units of nanomoles per litre (nmol/L) (except for lead, which was reported in micromoles per litre). The LOD varied from 0.4 to 3.0 nmol/L. Organochlorine compounds were measured in blood plasma and analyzed by gas chromatograph (Agilent 6890) coupled to an electron capture detector (EDC) (Agilent G2397A) and mass spectrometry detector (Agilent 5973 Network) with Agilent MSD Chem software. Concentrations were reported in units of ug/L and the LOD varied from 0.005 to 0.09 ug/L. Perfluoroalkyl substances were measured in blood plasma using a Waters Acquity Ultra Performance Liquid Chromatography coupled to Waters Quattro Premier XE mass spectrometer and Waters MassLynx software, E-453, operated in the multiple reaction monitoring mode with an “electrospray” ion source in the negative mode. Concentrations were reported in units of ug/L with LOD that varied from 0.1 to 0.3 μg/L.

The analytical accuracy and measurement precisions were evaluated through rigorous method validation programs (Health Canada, 2010). Quality control measures employed included using field blanks to confirm that the samples had not been contaminated, using blind replicate samples as indicators of the measurement precision, and using blind control samples as indicators for the analytical accuracy. For external quality control, inter-laboratory comparison studies were performed.

**References**.

Arbuckle, T.E., Davis, K., Marro, L., Fisher, M., Legrand, M., LeBlanc, A., Gaudreau, E., Foster, W.G., Choeurng, V., Fraser, W.D., MIREC Study Group, 2014. Phthalate and bisphenol A exposure among pregnant women in Canada–results from the MIREC study. Environ. Int. 68, 55–65.

Langlois, E., Saravanabhavan, G., Arbuckle, T.E., Giroux, S., 2014. Correction and comparability of phthalate metabolite measurements of Canadian biomonitoring studies (2007–2012). Environ. Int. 64, 129–133.

Health Canada. “Fifth Report on Human Biomonitoring of Environmental Chemicals in Canada”. *Government of Canada*, <https://www.canada.ca/en/health-canada/services/environmental-workplace-health/reports-publications/environmental-contaminants/fifth-report-human-biomonitoring.html#s5-1-1>. Accessed May 15 2024.

**Appendix B.** Descriptive statistics (complete case vs. incomplete cases)

| **Table 1.** Participant sociodemographic characteristics and mean birth weight (grams) of complete vs incomplete cases among MIREC study participants in Canada, 2008-2011. | | | | |
| --- | --- | --- | --- | --- |
|  | **Complete cases** | | **Incomplete cases** | |
|  | **n (%)** | **Birth weight (g)**  **Mean (SD)** | **n (%)** | **Birth weight (g)**  **Mean (SD)** |
| Total | 1565 (100) | 3453 (524) | 417 (100) | 3450 (572) |
| Race and ethnicity |  |  |  |  |
| Others | 276 (18) | 3355 (520) | 63 (22) | 3509 (562) |
| White | 1289 (82) | 3473 (523) | 228 (78) | 3240 (562) |
| Age |  |  |  |  |
| ≤24 | 79 (5) | 3440 (588) | 38 (14) | 3329 (675) |
| 25-29 | 366 (23) | 3456 (503) | 77 (28) | 3616 (498) |
| 30-34 | 564 (36) | 3451 (493) | 91 (33) | 3460 (509) |
| 35-39 | 444 (28) | 3462 (554) | 58 (21) | 3299 (675) |
| 40+ | 112 (7) | 3417 (582) | 15 (5) | 3505 (380) |
| Education levels |  |  |  |  |
| High school diploma or less | 117 (7) | 3373 (573) | 43 (15) | 3340 (572) |
| Some college, trade school,     or college diploma | 438 (28) | 3444 (550) | 100 (35) | 3444 (532) |
| Undergraduate degree | 586 (37) | 3485 (513) | 95 (33) | 3497 (580) |
| Graduate degree | 424 (27) | 3439 (501) | 51 (18) | 3398 (646) |
| Household income | |  |  |  |
| ≤ $20,000 | 58 (4) | 3370 (522) | 14 | 3247 (490) |
| $20,001 - $40,000 | 125 (8) | 3433 (568) | 27 (7) | 3498 (595) |
| $40,001 - $60,000 | 151 (10) | 3449 (553) | 36 (13) | 3498 (501) |
| $60,001 - $80,000 | 252 (16) | 3425 (522) | 34 (17) | 3693 (535) |
| $80,001 - $100,000 | 323 (21) | 3439 (522) | 35 (17) | 3455 (617) |
| >$100,000 | 656 (42) | 3481 (511) | 59 (29) | 3399 (572) |
| Smoking status | |  |  |  |
| Never | 965 (62) | 3446 (499) | 172 (59) | 3418 (630) |
| Current | 72 (5) | 3337 (696) | 33 (11) | 3431 (564) |
| Former | 428 (27) | 3492 (534) | 70 (24) | 3525 (399) |
| Quit during   Pregnancy | 100 (6) | 3426 (569) | 15 (5) | 3481 (584) |
| Parity |  |  |  |  |
| 0 | 677 (43) | 3406 (530) | 135 (46) | 3410 (621) |
| 1 | 641 (41) | 3492 (501) | 110 (38) | 3518 (550) |
| 2 | 186 (12) | 3486 (569) | 35 (12) | 3372 (494) |
| 3+ | 61 (4) | 3449 (530) | 11 (4) | 3500 (293) |
| Pre-pregnancy BMI |  |  |  |  |
| Underweight | 38 (2) | 3264 (473) | 11 (7) | 3445 (609) |
| Normal | 949 (61) | 3434 (491) | 91 (59) | 3377 (544) |
| Overweight | 341 (22) | 3516 (523) | 30 (20) | 3492 (509) |
| Obese | 237 (15) | 3465 (640) | 21 (14) | 3475 (740) |
| Infant Sex |  |  |  |  |
| Male | 831 (53) | 3508 (528) | 143 (50) | 3456 (567) |
| Female | 734 (47) | 3390 (514) | 143 (50) | 3451 (575) |

| **Table 2.** Participant hardship characteristics and mean birth weight (grams) of complete vs incomplete cases among MIREC study participants in Canada, 2008-2011. | | | | |
| --- | --- | --- | --- | --- |
|  | **Complete cases** | | **Incomplete cases** | |
|  | **n (%)** | **Birth weight (g)**  **Mean (SD)** | **n (%)** | **Birth weight (g)**  **Mean (SD)** |
| Total | 1565 (100) | 3452 (532) | 417 (100) | 3450 (572) |
| Types of Hardship | | | | |
| Racially minoritized |  |  |  |  |
| Yes | 276 (18) | 3355 (520) | 63 (22) | 3240 (562) |
| No | 1289 (82) | 3473 (523) | 228 (78) | 3509 (562) |
| Low income |  |  |  |  |
| Yes | 58 (4) | 3370 (522) | 14 (7) | 3247 (490) |
| No | 1507 (96) | 3456 (524) | 191 (93) | 3496 (569) |
| Low education |  |  |  |  |
| Yes | 117 (7) | 3374 (573) | 43 (15) | 3400 (572) |
| No | 1448 (93) | 3459 (520) | 246 (85) | 3455 (574) |
| Lone parenthood |  |  |  |  |
| Yes | 67 (4) | 3374 (552) | 20 (7) | 3481 (383) |
| No | 1498 (96) | 3456 (523) | 271 (93) | 3448 (549) |
| Living alone |  |  |  |  |
| Yes | 28 (2) | 3389 (590) | 8 (3) | 3363 (859) |
| No | 1537 (98) | 3454 (523) | 283 (97) | 3453 (563) |
| Current student |  |  |  |  |
| Yes | 158 (10) | 3440 (480) | 43 (11) | 3575 (502) |
| No | 1407 (90) | 3454 (529) | 356 (89) | 3437 (582) |
| Immigrant |  |  |  |  |
| Yes | 288 (18) | 3397 (535) | 335 (80) | 3293 (610) |
| No | 1277 (82) | 3465 (521) | 82 (20) | 3494 (555) |
| Low supplemental folic acid intake (<400 ug/day) | | |  |  |
| Yes | 74 (5) | 3371 (492) | 30 (7) | 3330 (619) |
| No | 1491 (95) | 3457 (526) | 387 (93) | 3461 (568) |
| Having chronic illnesses |  |  |  |  |
| Yes | 377 (24) | 3383 (580) | 114 (28) | 3507 (570) |
| No | 1188 (76) | 3475 (504) | 288 (72) | 3418 (575) |
